# The safety and efficacy of melatonin in the treatment of COVID-19: A systematic review and meta-analysis

**DOI:** 10.1097/MD.0000000000030874

**Published:** 2022-09-30

**Authors:** Xin-Chen Wang, Guang-Liang Wu, Ye-Feng Cai, Shi-Jie Zhang

**Affiliations:** a School of Food and Pharmaceutical Engineering, Zhaoqing College, Duanzhou District, Zhaoqing City, China; b Department of Neurology, The Second Affiliated Hospital of Guangzhou University of Chinese Medicine, Guangzhou, China; c Department of Neurology, Guangdong Provincial Hospital of Chinese Medicine, Guangzhou, China.

**Keywords:** COVID-19, SARS-CoV-2, melatonin, meta-analysis

## Abstract

As an infectious disease caused by the severe acute respiratory syndrome coronavirus 2 (SARS-CoV-2), the common signs of coronavirus disease 2019 (COVID-19) are respiratory symptoms, fever, cough, shortness of breath, and dyspnea, with multiple organ injuries in severe cases. Therefore, finding drugs to prevent and treat COVID-19 is urgently needed and expected by the public. Several studies suggested beneficial effects of melatonin for the relevant prevention and treatment. To explore the effect and safety of melatonin in the treatment and provide theoretical support and reference for seeking the most suitable drug for COVID-19, the meta-analysis was carried out accordingly.

**Methods:**

It included randomized clinical trials of patients with COVID-19 treated with melatonin. Total effective rate was the primary outcome, while C-reactive protein (CRP), arterial oxygen saturation (SaO_2_), white blood cell count (WBC) were the secondary measures. Random-effect and fixed-effect models were used to evaluate the effect size of some indicators in this meta-analysis.

**Results:**

Six eligible studies with 338 participants were included. One hundred seventy subjects were treated with melatonin adjuvant therapy and 168 subjects were assigned to the control group, with total excellent effective rate in subjects treated with melatonin [odds ratio = 3.05, 95 % confidence interval (CI) = 1.47, 6.31, *P* = .003]. Homogeneity was analyzed by fixed effect model (*I*^2^ = 0%). There was no significant difference in CRP between the melatonin group and the control group (weighted mean difference [WMD] = –0.36, 95% CI = –3.65, 2.92, *P* = .83). Significant difference was not existed in SaO_2_ between the melatonin treatment group and the control group (WMD = 1, 95% CI = –1.21, 3.22, *P* = .37). In terms of WBC, there was no significant difference between the 2 groups (WMD = –1.07, 95% CI = –2.44, 0.30, *P* = .13).

**Conclusions:**

The meta-analysis showed that melatonin had the beneficial effects for COVID-19 prevention and treatment as an adjunctive agent in combination with basic treatment for the treatment.

## 1. Introduction

The coronavirus pandemic disease 2019 (COVID-19) is caused by severe acute respiratory syndrome coronavirus 2 (SARS-COV-2) as a serious threat to global health security, making global countries and people suffered from the serious situations. All coronavirus can damage the respiratory tract, but SARS-COV-2 also affects the heart, gastrointestinal system, liver, kidneys, and central nervous system, ultimately leading to multiple organ failure.^[1,2]^ A recent study also showed that the human-to-human transmission rate of SARS-COV-2 was higher than the infection rate of the 2 previous coronavirus SARS-COV and middle east respiratory syndrome coronavirus.^[3]^ Signs and symptoms of COVID-19 patients are also varied at different stages, but common clinical symptoms include fever, fatigue, cough, shortness of breath, etc.^[4]^ Asymptomatic infected persons have uncommon symptoms, such as sore throat, headache, confusion, hemoptysis, etc,^[5]^ and some symptoms are mild or even have no typical symptoms at all.^[6]^ To date, the key management approaches to treating COVID-19 patients are early diagnosis, immediate isolation of patients, and prevented of infection.^[7]^ Typical treatment includes general supportive care, respiratory support, and nutritional support.^[8,9]^ The high infection rate and adverse consequences have brought great threats to the global economic development and human life quality. Seeking safe and effective treatment drugs has been the most urgent problem to be solved.

Melatonin is a methoxyindole hormone synthesized and secreted by the pineal gland of vertebrates at night under normal light/dark conditions. The main function is transmitting information about the circadian cycle to the body structure, coordinating the body behavior, and physiological functions to comply with the changes of the global environment, season, and circadian rhythm.^[10]^ Melatonin plays a key role in a variety of physiological activities, including the regulation of circadian rhythms, immune responses, oxidative processes, apoptosis, or mitochondrial homeostasis.^[11]^ Melatonin has a positive effect on acute respiratory stress caused by viruses, bacteria, and radiation,^[12]^ which can enhance the host tolerance to pathogen invasion, reduce the severity and mortality of fatal viral infections, including COVID-19 based on its antioxidant, anti-inflammatory, and immunomodulatory functions.^[13]^ Melatonin is considered to be an agent with extraordinary potential and efficacy during the treatment of COVID-19, which has been used in a number of randomized controlled trials (RCT).^[14]^

At present, some experimental studies have shown that melatonin could bring obvious effect in the treatment of COVID-19, but there are few studies on RCT. On this basis, the meta-analysis of the indicators in the currently published RCT related to COVID-19 was conducted, such as C-reactive protein (CRP), arterial oxygen saturation (SaO_2_), white blood cells (WBC), so as to evaluate the safety and efficacy of melatonin in the treatment, thus providing theoretical support for seeking suitable treatment scheme in the future.

## 2. Methods

### 2.1. Search strategy

After strictly abiding by the guidelines recommended in the Preferred Reporting Items for Systematic Reviews and Meta-analysis,^[15]^ it searched database Pubmed, Embase, Web of Science, Cochrane Library, and Clinical Trials.gov for all published literature from December 2019 to June 2022. The searching terms were “COVID-19” or “SARS-COV-2” or “SARS-CoV-2 Infection” or “COVID-19 Virus Disease” or “2019-nCoV Infection” or “Coronavirus Disease-19” and “Melatonin.” The language for searching literature was limited to Chinese and English, which was supplemented by a manual search on the list of references cited in the original and reviewed articles. If the study was published in peer-reviewed journals in English or Chinese, it would be included in the meta-analysis. After removing case studies, review articles, animal studies, brief exchanges, and letters to the editor, the search scope was limited to RCT of human studies. The study protocol was registered in PROSPERO (CRD42022306215).

### 2.2. Selection process

The EndNote 20 software was used to process the literature retrieval records by applying 2 reviewers (X.C.W. and G.L.W.) in the same criteria for each study, extracting data separately from the included papers, so as to collect the following information: Author and year, gender, sample size, age, intervention, duration, and outcome measures. If there are objections, consensus would be reached by reviewing the original report and discussing further with the 3rd reviewer (S.J.Z).

Inclusion criteria: COVID-19 was diagnosed by chest X-ray or CT imaging, clinical symptoms, reverse transcription polymerase chain reaction nasopharynx swab detection; positive subjects were patients with COVID-19 aged over 18 years; melatonin was used in the treatment group; all included studies were RCT.

Exclusion criteria: The subjects were not human experiments; review articles, cell experiments; melatonin was used in the control group; COVID-19 related indicators were excluded; the data were incomplete.

### 2.3. Data extraction

The 2 reviewers independently extracted the following contents, including the first author, year of publication, country, average age, gender, sample size, diagnostic criteria, intervention measures, treatment cycle, and COVID-19 related indicators, such as CRP, arterial oxygen tension, inspired oxygen fraction, SaO_2_, and WBC. At the end of the study, the data were presented in the form of mean ± standard deviation, the Cochrane Handbook was used to evaluate the bias of RCT included in the analysis.^[16]^ On this basis, it evaluated the blinding result evaluation, selective reporting, incomplete result data, and other measures of participants and researchers, such as the generation of deviation risk series and allocation concealment in random samples.

### 2.4. Outcome measures

Without extracting the relevant results of each study in the meta-analysis, it only extracted and analyzed the cross results involved in RCTs. The primary outcome measures were clinical recovery rate, and the secondary outcome measures were CRP, SaO_2_, and WBC.

### 2.5. Statistical analysis

Review Manager 5.4 software was applied to conduct meta-analysis. The Cochrane Q test and *I*^2^ statistic were involved to assess the extent of heterogeneity during the analysis. If the *I*^2^ statistic reached or exceeded 50%, indicating the heterogeneity, and applying the random effect model. Otherwise, the fixed effect model would be adopted.^[17]^ The analysis of continuous data in the meta-analysis could be denoted as a standard mean difference with a 95% confidence interval (CI), while the dichotomous data could be implied by an odds ratio with a 95% CI.^[18]^ At the time of analysis, *P *< .05 was treated as statistically significant.

## 3. Result

### 3.1. Search results

**Figure 1.**
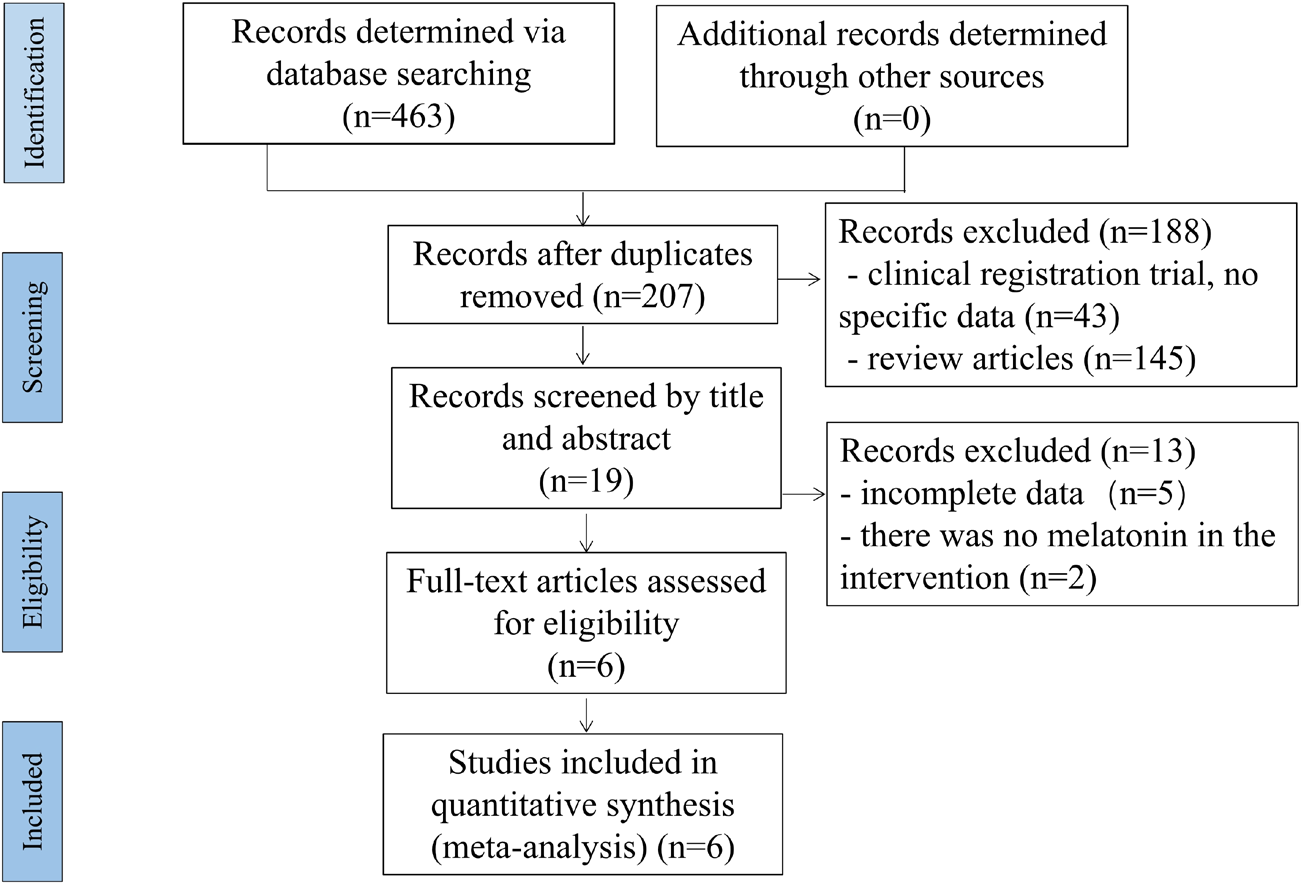
Systematic reviews and meta-analysis flow chart of the literature search.

Based on the retrieval strategy and screening method, 463 records were found from database. According to the pre-set selection criteria, 457 articles were excluded, including 256 duplicate records, 41 without detailed clinical data, and 160 studies of review type or not met the inclusion criteria (Fig. 1). Finally, 6 were included in this study,^[19–24]^ as shown in Table 1.

**Table 1:** Characteristics of studies included in the meta-analysis.

Author, year	Country	Patients (no. I/C)	Age years, range(mean ± SD), I/C	Male/Female (no.I/C)	Intervention	Control	Treatment duration (days)	Outcome measurement	ICU/Not
Zahra Alizadeh, 2021^[19]^	Iran	14/17	21–60, 37.57 ± 8.2/ 34.53 ± 8.2	9/5,8/9	Melatonin (6 mg/d) was consumed half an hour before bedtime every night in low light conditions + Regular medication (if need, hydroxychloro-quine, acetaminophen, and naproxen)	Regular medication (if need, hydroxychloro-quine, acetaminophen, and naproxen)	14	CRP	ICU
Mahboubeh Darban, 2021^[20]^	Iran	10/10	18–65, NA	NA	IV vitamin C (2g, q6 hr), oral melatonin (6 mg, q6 hr), and oral zinc sulfate (220 mg containing 50 mg elemental zinc, q6hr) + Azithromycin (250 mg/d), lopinavir/ritonavir (100 mg/25mg/day), glucocorticoids, and necessary oxygen.	Azithromycin (250 mg/d), lopinavir/ritonavir (100mg/25mg/d), glucocorticoids, and necessary oxygen.	10	PaO_2_/FiO_2_, SaO_2_, LDH, WBC, Lymph, ESR, CRP	ICU
Gholamreza Farnoosh, 2021^[21]^	Iran	24/20	18 above， 50.75 ± 14.43/ 52.95 ± 14.07	14/10, 12/8	Melatonin (3 mg 3 times/d) + Regular medication	Regular medication	14	NLR, ESR, CRP	ICU
Carolina Bologna, 2021^[22]^	Iran	40/40	NA, 71.6 ± 7.7/ 71.8 ± 8.8	23/17, 23/17	Melatonin (2 mg/d)	Without therapy	7	GOT, GPT, CRP	ICU
Seyed Abbas Mousavi, 2021^[23]^	Iran	48/48	NA, 51.06 ± 15.86/ 54.77 ± 15.34	25/23, 18/30	Melatonin (3 mg/d) + Regular medication	Regular medication	10	WBC, Lymph, CRP, SaO_2_	ICU
Nafifiseh Alizadeh，2022^[24]^	Iran	34/33	18 above, 61.27 ± 18.09/ 65.35 ± 19.30	19/15, 24/9	Melatonin (21mg/d) + Remdesivir (200 mg on the first day and 100 mg daily for 4 days thereafter), corticosteroids, anticoagulant (prophylactic dose), and sometimes tocilizumab	Remdesivir (200 mg on the first day and 100 mg daily for 4 days thereafter), corticosteroids, anticoagulant (prophylactic dose), and sometimes tocilizumab	6	WBC, Cr, ALT, AST, CRP, ESR	ICU

ALT = alanine aminotransferase, AST = aspartate aminotransferase, Cr = serum creatinine, CRP = C-reactive protein, ESR = erythrocyte sedimentation rate, FiO_2_ = inspired oxygen fraction, GOT = glutamate oxaloacetate transaminase, GPT = glutamate pyruvate transaminase, ICU = intensive care unit, LD = lactate dehydrogenase, Lymph = lymphocytes count, NLR = neutrophil-lymphocyte ratio, PaO_2_ = arterial oxygen tension, q6 hr = every 6 hours, Regular medication = Regarding the Iranian national COVID-19 treatment protocol, regular medication included oxygen therapy, conservative rehydration and empirical antibiotic therapy, SaO_2_ = oxygen saturation, WBC = white blood cells.

### 3.2. Risk of bias assessment

**Figure 2.**
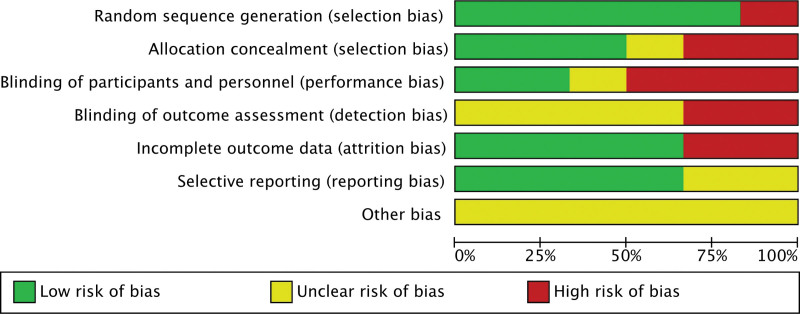
Assessment of risk of bias for the studies.

**Figure 3.**
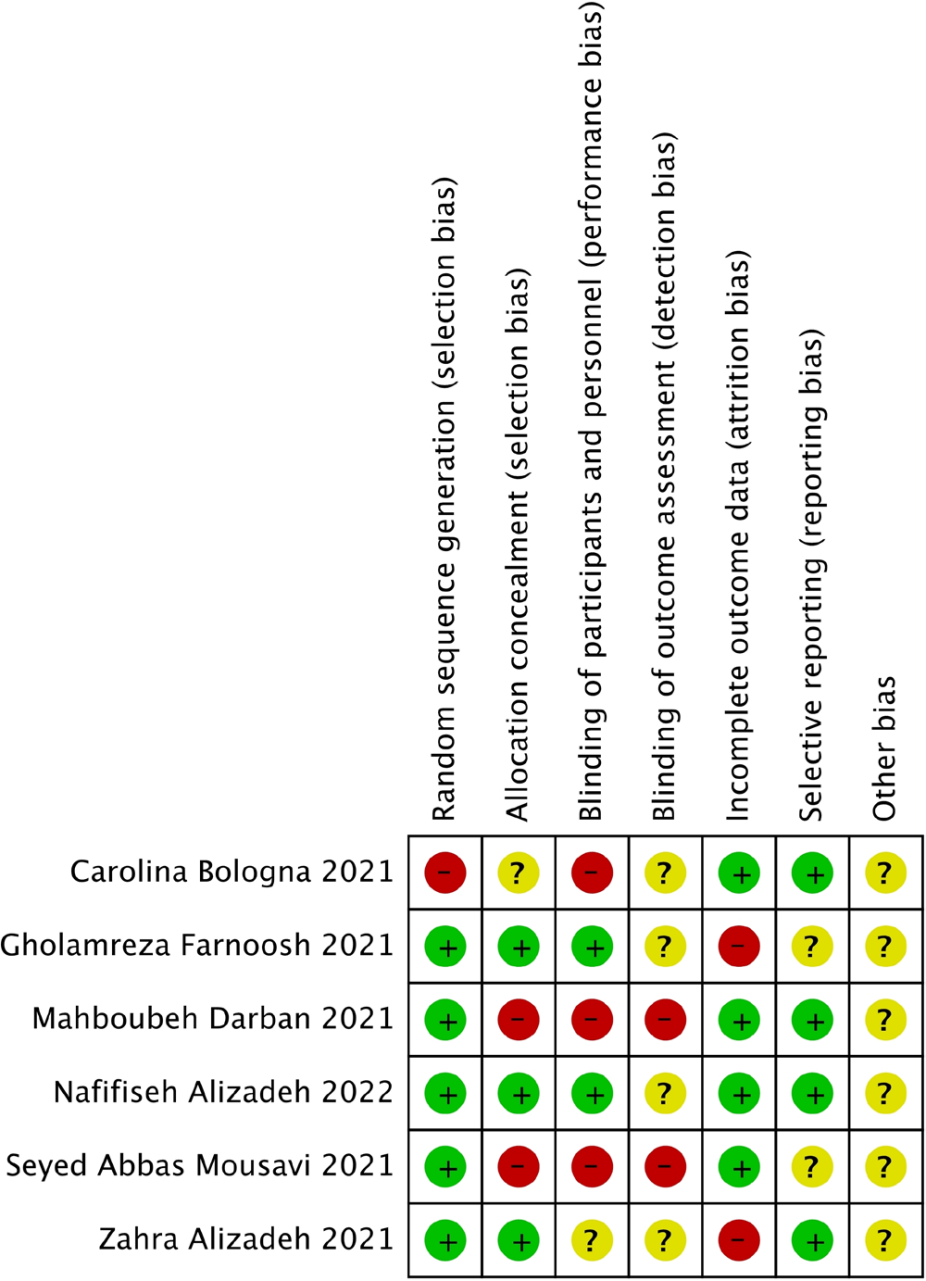
Assessment of risk of bias for the studies.

The assessment of the risk of bias was illustrated in Figures 2 and 3. The 6 included literatures^[19–24]^ were all single-center studies, and 5 studies^[19–21,23,24]^ were conducted in regional hospitals in Iran. Among them, 2 articles^[20,23]^ mentioned random double blindness, 1 literature^[19]^ mentioned random single blindness, 1 study^[20]^ used active-control, open-label, parallel-group, and 1 article^[22]^ did not mention random blindness. One study^[23]^ was an investigator-initiated, open-label, randomized parallel group, and actively controlled clinical trial. The melatonin groups in all studies were given melatonin except for the standard treatment. Zahra Alizadeh^[19]^ used 6 milligram (mg)/day melatonin as intervention measure. Mahboubeh et al^[20]^ used a higher dose of melatonin of 6 mg/6 hours in the intervention group. Gholamreza et al^[21]^ gave melatonin at a dose of 3 mg 3 times a day at large basis. Two studies^[22,23]^ were given standard doses of 2 to 3 mg/day. One study^[23]^ utilized a high-dose melatonin 21 mg/day as intervention measure.

### 3.3. Primary outcome

**Figure 4.**
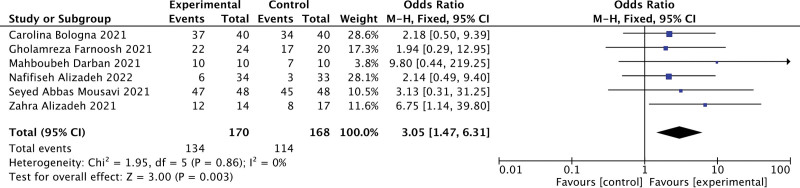
Forest plots showing OR with 95% CI for the total effective rate comparing treatment with or without MT in a fixed-effect model. CI = confidence interval, MT = melatonin, OR = odds ratio.

**Figure 5.**
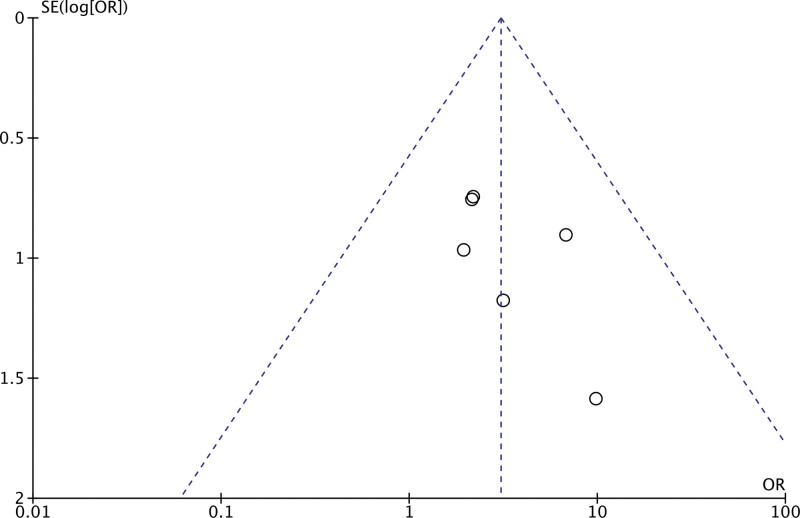
Funnel plot of overall clinical efficacy.

In the 6 trials, 170 subjects were treated with melatonin adjuvant therapy and 168 subjects were assigned to the control group. The control group was given conventional antiviral drugs, while the treatment group was given melatonin except for conventional treatment. As shown in Figure 4, the total effective rate was higher in subjects treated with melatonin (odds ratio = 3.05, 95% CI = 1.47, 6.31; *P *= .003). Homogeneity was analyzed by fixed effect model (*I*^2^ = 0%), and symmetrical funnel plots showed no potential publication bias (Fig. 5).

### 3.4. Secondary outcomes

**Figure 6.**
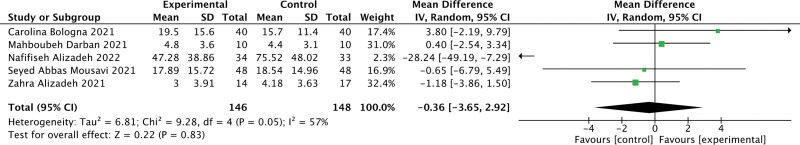
Forest plots showing WMD with 95% CI for changes of C-reactive protein (CRP) comparing treatment with or without MT in a fixed-effect model. CI = confidence interval, MT = melatonin, WMD = weighted mean difference.

**Figure 7.**
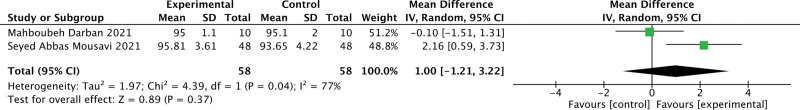
Forest plots showing WMD with 95% CI for changes of arterial oxygen saturation (SaO_2_) comparing treatment with or without MT in a random-effect model. CI = confidence interval, MT = melatonin, WMD = weighted mean difference.

**Figure 8.**
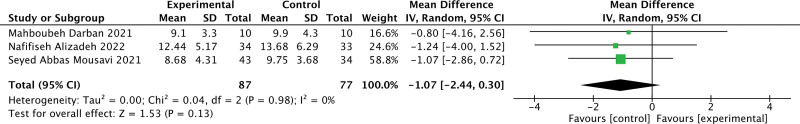
Forest plots showing WMD with 95% CI for changes of white blood cell (WBC) comparing treatment with or without MT in a random-effect model. CI = confidence interval, MT = melatonin, WMD = weighted mean difference.

Four studies^[19,20,22–24]^ mentioned changes in CRP after melatonin administration, and there was no significant difference in CRP between the melatonin group and the control group (weighted mean difference [WMD] = –0.36, 95%CI = –3.65, 2.92, *P *= .83) (Fig. 6). There was no significant difference in SaO_2_ between the melatonin treatment group and the control group in the 2 studies^[20,23]^ (WMD = 1, 95% CI = –1.21,3.22, *P *= .37) (Fig. 7). In the 3 studies,^[20,23,24]^ there was no significant difference in WBC count between the melatonin treated group and the control group (WMD = –1.07, 95% CI = –2.44, 0.30, *P* = .13) (Fig. 8).

### 3.5. Sensitivity analysis

Due to the good homogeneity of the primary outcome (*I*^2^ = 0%), a sensitivity analysis of the overall clinical efficacy was not conducted.

### 3.6. Adverse events

A total of 338 subjects were enrolled in 6 studies. One study did not mention the occurrence of adverse reactions.^[20,24]^ Three studies^[19,21,22]^ reported no adverse reactions during melatonin use. One study^[23]^ indicated that 1 patient died in the intervention group.

## 4. Discussion

Three hundred thirty eight subjects in 6 studies were included for the analysis when comparing the recovery rate, CRP, SaO_2_, WBC, and adverse events between the melatonin intervention group and the control group. The improvement rate of the melatonin intervention group was significantly higher than that of the control group, and the improvement of COVID-19 related indicators was also better than that of the control group. There was no heterogeneity beyond random error in CRP and WBC number (*I*^2^ = 0 %), and there was heterogeneity in SaO_2_ (*I*^2^ = 77%), which might be due to the small sample size of clinical subjects and poor test efficiency.

Melatonin proved as a potential drug for the treatment of various sleep disorders in adults, without tolerance risk, dependence, or suspension effects for exogenous melatonin. It not only had the least side effects, but also brought short half-life.^[25]^ Tablets such as general drug dosage form, the standard dosage shall be 2mg/day, and taken orally 1 to 2 hours before bed in a dark environment. The treatment duration varied from 7 days to 24 weeks.^[26,27]^ After consulting a large number of literature and guideline, no relevant study on the relationship between melatonin dose selection and patient weight was found.

In recent years, some studies began to focus on the effect of melatonin on COVID-19. In 1 study,^[20]^ the melatonin intervention group was treated with vitamin C and magnesium sulfate in combination with a high dose of melatonin (6 mg/6 hours). Some studies have suggested that although the recognized safe dosage range of melatonin is 2 to 10 mg, only the dosage above 10 mg can be effective in animal experiments.^[28]^ Schrire et al^[29]^ conducted a meta-analysis of adverse reactions in the treatment of various diseases with dosage of more than 10 mg of melatonin, then found that high doses of melatonin showed a good safety. Nafiseh et al^[23]^ applied 21 mg high-dose melatonin as adjunctive therapy to COVID-19 critically patients. The results showed that melatonin did not significantly change the death outcome, but the CRP level was significantly improved compared to the control group (*P *= .042).

However, further researches and explorations are needed due to limited research evidences and clinical data. In addition, trace elements may play a fundamental role in disease susceptibility and maintenance of immune system function.^[30]^ Whether high doses of melatonin combined with vitamin C and magnesium sulfate could increase the therapeutic effect of melatonin or reduce its adverse reactions remained to be confirmed by further studies.

Two studies^[22,23]^ mentioned the improvement of sleep in patients with COVID-19 by melatonin, who suffer from night and day reversal, disturbed sleep, and even delirium in the later stages of treatment due to isolation. Melatonin can be released into the blood at night in accordance with the circadian rhythm to improve the sleep quality of insomnia patients by activating MT1 and MT2 receptors.^[31]^ Compared to other sleep drugs, it is well tolerated and has low dependence.^[32]^ Total sleep duration and delirium episodes were analyzed during the study (*P *< .001).^[22]^ Comparing the indicators of getting into sleep, sleep quality, awakening status, feeling following wakefulness from the 1st day to the 7th day of melatonin treatment, it was found that all had statistical significance in the study^[23]^ (*P < *.001). It is vital to carry out further research to determine whether melatonin can effectively inhibit delirium in patients with COVID-19. The melatonin doses used in the 2 studies^[22,23]^ were 2 and 3 mg, respectively. To control the drug release rate and reduce or avoid the “peak valley” fluctuation of blood drug concentration, melatonin was made into a prolonged-release agent. Carolina et al^[22]^ applied in the study, a dose of 2 mg of prolonged-release melatonin (PRM) through the gradual release of melatonin and used for melatonin receptor to simulate the physical release of melatonin. Since PRM can effectively improve sleep quality, reduce the onset of delirium, promote patient adherence to ventilation, then generally enhance the prognosis.^[33]^ It is crucial to explore the issues of whether the PRM is different from regular melatonin in improving sleep quality and reducing delirium episodes in COVID-19 patients, or whether it will be more secure.

In view of the current research status of melatonin treatment of COVID-19, it suggests to conduct open, randomized parallel group and active controlled clinical trials initiated by researchers. Therefore, with the following ideas for future experimental research, melatonin acts as one of the commonly used supplements for the treatment of jet lag, delayed sleep wake disturbance, and insomnia, with a lower dosage of 0.5 to 5 mg.^[34]^ Two milligrams is the commonly used dose for most melatonin treatment of COVID-19 at present. Six milligrams is the higher dose in existing studies. Some studies have found that although having high dose above 10 mg, it still owns certain safety.^[29]^ In this experiment, it suggests that the set dose of melatonin shall be controlled to 2, 6, and 10 mg. It requires large sample size urgently, whose number of patients shall be included at least 100 to 120 or more, so as to record the clinical symptoms, such as fever, cough, dyspnea, chest pain, gastrointestinal disorders, headache, and some index SaO_2_, arterial oxygen tension, inspired oxygen fraction, total sleep duration, delirium times, thus studying the effectiveness and safety of different melatonin doses in the treatment of COVID-19.

It has been found that melatonin can reduce the generation of oxygen species and free metal ions,^[35]^ prevent harmful conditions, such as DNA damage, protein oxidation, and lipid peroxidation,^[36]^ inhibit oxidative stress and cell apoptosis to prevent lung inflammation caused by COVID-19,^[37]^ with higher antioxidant capacity than other active oxygen scavengers.^[38]^ Although it has diverse effects of COVID-19 and different mutant strains (Alpha, Beta, Gamma, Delta, and Omicron) on the host, melatonin owns certain inhibitory and blocking effects in the process of inhibiting virus replication and maturation. Therefore, it believes that it has positive effect of melatonin on COVID-19 and its variants, but still needs to carry on long-term research for verification.

With certain limitations, most of the studies were conducted in regional hospitals in Iran, which were single centers with limited geographical distribution. The small sample size and limited ability of systematic review may lead to publication bias. In this meta-analysis, it selected only a few indicators for comparison without significant difference between the intervention group and the control group, which was related to the small number of studies, but the results were all of low heterogeneity. Three studies^[19,20,24]^ mentioned that improvement was based on the alleviation of symptoms and indicators. In 2 studies,^[21,23]^ the patients discharged from hospital and survival were used as improvement criteria. In 1 study,^[22]^ removal from quarantine and no deaths were used as criteria for improvement. Due to the different improvement criteria mentioned in the 6 studies, it could not define and analyze the improvement rate by a uniform standard.

The results suggest that melatonin is effective in improving symptoms, which reduces adverse reactions, and improves life quality when used in combination with conventional medications for the treatment of COVID-19. However, multi-center, large sample, and well-designed RCT are needed to further explore the role of melatonin due to the limited research content.

## 5. Conclusion

Based on the urgency of the current COVID-19 epidemic, it is urgent to find a drug for prevention and treatment. The results show that melatonin can improve the relevant symptoms and indicators of COVID-19 with certain safety, which provides a theoretical basis for exploring the treatment in the future.

## Author contributions

X.W. finished the data analysis. X.W., G.W., and S.Z. wrote and revised the manuscript. S.Z. conceived and designed the study. Y.C. and S.Z. acquired funding supports and approved the final manuscript as submitted.

**Formal analysis:** Xin-Chen Wang, Shi-Jie Zhang.

**Investigation:** Xin-Chen Wang, Guang-Liang Wu, Shi-Jie Zhang.

**Project administration:** Xin-Chen Wang, Ye-Feng Cai.

**Resources:** Xin-Chen Wang, Guang-Liang Wu, Shi-Jie Zhang.

**Software:** Xin-Chen Wang, Guang-Liang Wu.

**Supervision:** Ye-Feng Cai, Shi-Jie Zhang.

**Visualization:** Ye-Feng Cai.

**Writing – review & editing:** Xin-Chen Wang.
